# Preparation of aripiprazole-poly(methyl vinyl ether-*co*-maleic anhydride) nanocomposites via supercritical antisolvent process for improved antidepression therapy

**DOI:** 10.1093/rb/rbac080

**Published:** 2022-10-12

**Authors:** Lin-Fei Chen, Ying Chen, You-Yu Duan, Man-Man Zhang, Pei-Yao Xu, Ranjith Kumar Kankala, Shi-Bin Wang, Ai-Zheng Chen

**Affiliations:** College of Chemical Engineering, Huaqiao University, Xiamen 361021, PR China; Institute of Biomaterials and Tisszue Engineering, Huaqiao University, Xiamen 361021, PR China; Fujian Provincial Key Laboratory of Biochemical Technology, Huaqiao University, Xiamen 361021, PR China; College of Chemical Engineering, Huaqiao University, Xiamen 361021, PR China; Institute of Biomaterials and Tisszue Engineering, Huaqiao University, Xiamen 361021, PR China; Fujian Provincial Key Laboratory of Biochemical Technology, Huaqiao University, Xiamen 361021, PR China; College of Chemical Engineering, Huaqiao University, Xiamen 361021, PR China; Institute of Biomaterials and Tisszue Engineering, Huaqiao University, Xiamen 361021, PR China; Fujian Provincial Key Laboratory of Biochemical Technology, Huaqiao University, Xiamen 361021, PR China; College of Chemical Engineering, Huaqiao University, Xiamen 361021, PR China; College of Chemical Engineering, Huaqiao University, Xiamen 361021, PR China; Institute of Biomaterials and Tisszue Engineering, Huaqiao University, Xiamen 361021, PR China; Fujian Provincial Key Laboratory of Biochemical Technology, Huaqiao University, Xiamen 361021, PR China; College of Chemical Engineering, Huaqiao University, Xiamen 361021, PR China; Institute of Biomaterials and Tisszue Engineering, Huaqiao University, Xiamen 361021, PR China; Fujian Provincial Key Laboratory of Biochemical Technology, Huaqiao University, Xiamen 361021, PR China; Institute of Biomaterials and Tisszue Engineering, Huaqiao University, Xiamen 361021, PR China; Fujian Provincial Key Laboratory of Biochemical Technology, Huaqiao University, Xiamen 361021, PR China; College of Chemical Engineering, Huaqiao University, Xiamen 361021, PR China; Institute of Biomaterials and Tisszue Engineering, Huaqiao University, Xiamen 361021, PR China; Fujian Provincial Key Laboratory of Biochemical Technology, Huaqiao University, Xiamen 361021, PR China

**Keywords:** aripiprazole, supercritical fluid technology, antidepression, new nano-formulation

## Abstract

Aripiprazole (ARI), a second-generation atypical antipsychotic drug approved for schizophrenia treatment, shows good efficacy against depression. However, the poorly aqueous solubility of ARI leads to low bioavailability and increased dose-related side effects, seriously limiting its application in pharmaceutics. Herein, we demonstrated the fabrication of ARI and poly (methyl vinyl ether-*co*-maleic anhydride) (PVMMA) composite nanoparticles (PA NPs) using the supercritical antisolvent (SAS) process for enhancing its water-solubility and curative anti-depressant effects. Initially, the optimal experimental conditions (ARI/PVMMA mass ratio of 1:6, pressure of 10 MPa, and solution flow rate of 0.75 ml min^−1^) were determined by a 2^3^ factorial experimental design, resulting in the PA NPs with an excellent particle morphology. *In vitro* cell experiments showed that PA NPs significantly inhibited the inflammatory response caused by the microglia activation induced by lipopolysaccharide (LPS). Similarly, mice behavioral tests demonstrated that PA NPs significantly improved LPS-induced depression-like behavior. Importantly, compared with free ARI, the LPS-induced activation of microglia in the mouse brain and the expression of inflammatory factors in serum were significantly reduced after treatment with PA NPs. Together, the innovative PA NPs designed by SAS process might provide a candidate for developing new ARI-based nano-formulations.

## Graphical abstract

**Figure:**
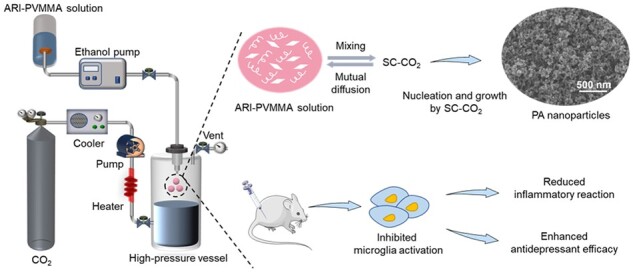

## Introduction

Depression is a central nervous system disease, which often shows the characteristics of loss of interest in activities, lack of energy and courage, irregular sleep patterns, as well as appetite disorders, even leading to suicide in severe cases [1]. Currently, depression has become a problem endangering people’s health worldwide and is expected to become one of the three contributors to the global disease burden by 2030 [2]. Therefore, developing effective treatment strategies to overcome depression is of great significance.

Among various hypotheses regarding pathogenesis, neuroinflammation has become a hot spot in the study of depression [3, 4]. The overexpression of inflammatory factors and neurotoxins caused by microglial activation is closely related to the neuroinflammatory response [5]. Specifically, microglia, the immune cells of the nervous system, are activated during neuroinflammation triggered by depression, resulting in the overexpression of various pro-inflammatory mediators, including tumor necrosis factor-α (TNF-α), interleukin-6 (IL-6) and NO, among others [6, 7]. Therefore, inhibiting microglia activation and anti-neuroinflammatory are considered effective treatment strategies for depression.

Aripiprazole (ARI), a second-generation or atypical antipsychotic, possesses excellent efficacy on negative symptoms of schizophrenia and major depressive disorders [8]. Previous studies indicated that ARI could inhibit the activation of microglia and correspondingly reduce the inflammatory cytokines secreted by pro-inflammatory microglia [9, 10]. Sato-Kasai *et al*. [11] demonstrated that ARI might inhibit the activation of microglia and exercise its anti-inflammatory activity by regulating the intracellular Ca^2+^ level *via* the transient receptor potential melastatin 7 (TRPM7) channels. The results from Racki *et al*. [12] also showed ARI might promote the transformation of microglia to an anti-inflammatory type and balance the level of cell metabolism by inhibiting mTORC1 activity. Considerably, ARI offers an excellent prospect for treating depression by inhibiting the anti-inflammatory response, such as microglia activation. However, the poor aqueous solubility of ARI leads to a low bioavailability issue, significantly limiting its therapeutic efficacy on depression and the development of new ARI-based formulations [13].

Recently, various ARI nano-formulations, including nanosuspensions [14], solid lipid nanoparticles [15] and polymeric micelles [16], have been developed to enhance their aqueous solubility and corresponding therapeutic efficacy. In particular, some studies presented that the solubility of water-insoluble drugs could be greatly improved by coprecipitating with some polymers that could promote drug nucleation into nano-sized particles [17, 18]. Poly(methyl vinyl ether-*co*-maleic anhydride) (PVMMA), a generally recognized biocompatible hydrophilic copolymer, has been widely used as a drug carrier to deliver hydrophobic drugs due to its unique advantages such as easy co-precipitation with drug molecules and promoting their nucleation [19, 20]. Importantly, various PVMMA-based drug delivery systems have been designed for oral [21], transdermal [22], nasal [23] and other modes of administration. These designs displayed excellent biosafety. In addition, PVMMA showed unique advantages in maintaining or enhancing the efficacy of active ingredients, and has been widely used in the delivery of anticancer drugs [24], immunosuppressants [25, 26] and proteins [27], among others.

In biomedical applications, an ideal active drug should satisfy both high therapeutic effect and low side effects, presenting higher requirements for the bioavailability of these drugs [28, 29]. The development of nanotechnology provides more strategies to solve the problem of poor bioavailability of insoluble drugs [30–32]. In recent years, nanocarriers have been widely used to deliver active pharmaceutical ingredients to improve the corresponding efficacy due to their high loading capacity, prevention of premature drug leakage, and ease of surface modification [33, 34]. Specially, nano-sized water-insoluble drugs have a larger specific surface area in contact with the surrounding aqueous medium, significantly promoting the improvement of their solubility and bioavailability. Although various nanonization methods, including spray-drying [35, 36], mechanical crushing [37], solvent evaporation [38] and nanoprecipitation [39], have been developed, their preparation methods often suffer from notable limitations, including cumbersome preparation process, high-temperature reaction conditions, and high residual organic solvents, among others [40]. Supercritical fluid (SCF) technology, an environmentally friendly process, has garnered increasing attention over various traditional technologies in drug nanonization due to simple operation, high controllability and low residual organic solvent [41–43]. In addition, as one of the most commonly used SCFs, supercritical carbon dioxide (SC-CO_2_) possesses mild and manageable critical conditions, in addition to other advantages, such as being environmentally friendly and economical [44, 45]. Among various SCF-based techniques, the supercritical antisolvent (SAS) process has great application potential in the preparation of the nano dosage forms of drugs [46], genes [47] and proteins [48]. In the SAS process, organic solvents (acetone, methanol and dichloromethane) with high drug solubility and SC-CO_2_ with low drug solubility diffuse each other, resulting in a high degree of supersaturation. Finally, drug molecules precipitate in the form of particles [49, 50]. In addition, the drug particles in the SAS process can be highly regulated by adjusting the parameters that significantly affect the particle morphology, including pressure, temperature, drug concentration and flow rate.

Based on these considerations, we demonstrate the fabrication of ARI and PVMMA composite nanoparticles (PA NPs) using the SAS process to enhance the water-solubility and bioavailability of ARI and the corresponding anti-depressant efficacy. To obtain the optimal formulation, we estimate the effects of drug/polymer (ARI/PVMMA) mass ratio, pressure, and solution flow rate on the nanoparticle morphology by a 2^3^ full-factorial experiment. Furthermore, *in vitro* drug dissolution experiments showed that the solubility of ARI in PA NPs was significantly increased, which could be attributed to their smaller size and amorphous physical by various characterization analyses. Finally, the anti-depressant effect of PA NPs by inhibiting inflammatory response was verified at the cellular and animal levels.

## Materials and methods

### Materials

ARI (purity ≥95%) was obtained from Energy Chemical Co., Ltd. (Shanghai, China). Tween 80, potassium bromide (KBr), poly (methyl vinyl ether-*co*-maleic anhydride) PVMMA and lipopolysaccharide (LPS) were obtained from Aladdin Co., Ltd. (Shanghai, China). Acetone was purchased from Sinopharm Chemical Reagent Co., Ltd. (Shanghai, China). Mouse TNF-α and IL-6 enzyme-linked immunosorbent assay (ELISA) kits were purchased from FANKEL Industrial Co., Ltd. (Shanghai, China). Griess reaction and CCK-8 assay kits were purchased from Beyotime Biotechnology Co., Ltd. (Shanghai, China).

### Preparation of PA NPs

**Figure 1.**
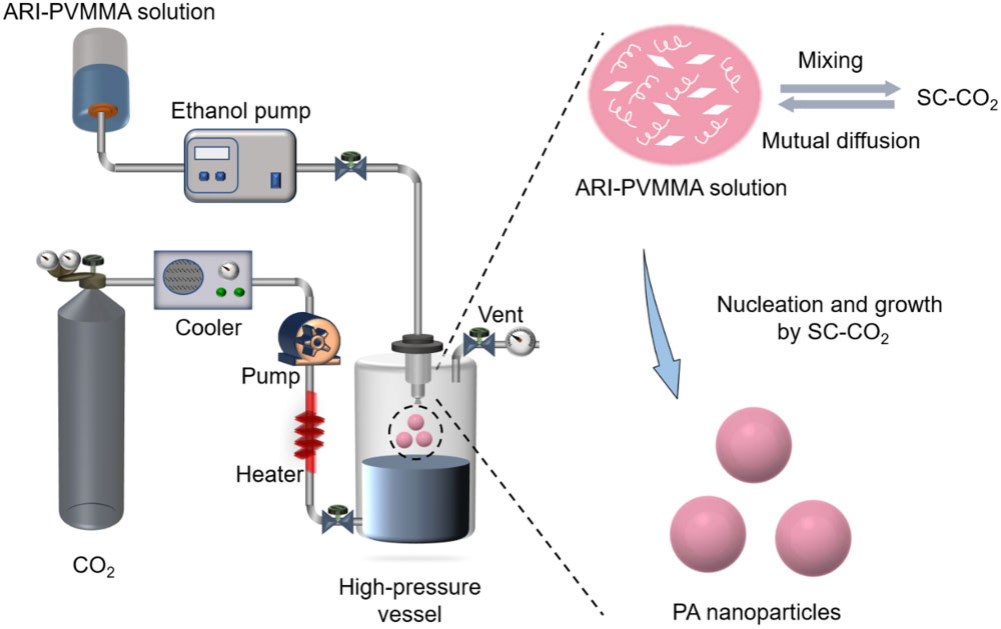
Schematic diagram of PA NPs prepared by the SAS process for antidepression therapy.

In this work, PA NPs were prepared by the SAS process. The schematic diagram of the SAS device (Waters, Massachusetts, USA) was shown in Fig. 1. Initially, CO_2_ was rapidly cooled to around 3°C through a low-temperature thermostat condensation system and then transported to the high-pressure crystallization vessel (HPV, 500 ml), adjusting the pressure and temperature according to the experimental design at the supercritical state of CO_2_ and remained constant. Then, 20 mg of ARI and 120 mg PVMMA were dissolved in 20 ml ACE, resulting in a homogeneous mixture solution. In these cases, the ARI/PVMMA mixture solution was then pushed into the HPV through an ethanol pressure pump at a certain flow rate. In HPV, the solution mixture was uniformly dispersed into SC-CO_2_ with high fluidity and high diffusivity through a special coaxial double-layered nozzle (inner diameter of 0.15 mm), and was fully mixed and diffused with each other, finally reaching a homogeneous phase. Furthermore, the SC-CO_2_ was pumped continuously for a further 20 min to remove the residual organic solvent from the sample. Finally, the HPV was gradually depressurized to ambient pressure, and the resulting PA NPs were collected and stored in a vacuum-drying oven for subsequent experiments. To further optimize the experimental results, various groups of PA NPs samples were prepared according to the 2^3^ full-factorial experimental design (see Supplementary Table S1) and stored for future use.

### Characterizations

The scanning electron microscopy (SEM) images of PA NPs were obtained from S4800 equipment (HITACHI, Tokyo, Japan). The chemical interactions between ARI and PVMMA in PA NPs were evaluated by the Fourier transform infrared (FT-IR) spectrophotometer (Nicolet iS50, Thermo Fisher Scientific, Waltham, USA). Differential scanning calorimeter (DSC) 200F3 (NETZSCH, Bavaria, Germany) was used to determine the thermal behavior of various samples, including free ARI, pure PVMMA and PA NPs. X-ray diffraction analyzer (XRD, SmartLa, Rigaku, Tokyo, Japan) was used to evaluate the crystallization behaviors of free ARI, pure PVMMA, physical mixture of ARI and PVMMA and PA NPs. Ultraviolet-visible (UV-vis) spectrophotometer (UV-1800, Shimadzu, Kyoto, Japan) was used to determine the drug loading efficiency of ARI in PA NPs.

### *In vitro* dissolution study

The drug dissolution of unprocessed ARI and PA NPs was recorded by incubating samples in phosphate buffered saline (PBS), mimicking the physiological (blood) microenvironments. Briefly, the samples with the same amount of ARI placed in the cellulose dialysis membrane (MWCO = 1.0 kDa) were dipped into a certain amount (25 ml) of PBS (pH-7.4, 37°C) and constantly stirred at 120 rpm. Then, the release medium (2 ml) was collected at predetermined time points (0.5, 1, 2, 4, 6, 8, 12, 18 and 24 h), and an equal amount of fresh PBS was rapidly replenished. Finally, the absorbance values at 254 nm were measured using a UV-vis to determine the cumulative ARI release at each time point. The cumulative drug release amount at time *n* ($M_{n}$)was calculated as:

$$M_{n}=C_{n}\times V+\sum C_{n-1}V_{s},$$

where $C_{n}$ is the concentration of ARI in the release medium at time *n*, *V* is the total volume of the release medium and $V_{s}$ is the volume of each sample that was collected for measurement. Finally, cumulative ARI release ratio was calculated following the formula:

$$\text{Cumulative ARI release (\%)}=\frac{M_{n}}{M_{\mathrm{total}}}\times 100,$$

where $M_{\mathrm{total}}\;$is the total amount of ARI encapsulated in the PA NPs.

### *In vitro* cellular investigations

#### Cell culture

Mouse microglia cells (BV-2) cells were cultured in the culture flasks with an adequate Dulbecco’s modified Eagle medium (DMEM, Grand Island, USA) containing 10% (v/v) FBS and 1% (v/v) antibiotics and cultured in an incubator (37°C, 5% CO_2_).

#### Cytotoxicity on BV-2 cells

The cytotoxicity of LPS, free ARI and PA NPs on BV-2 cells was determined by the CCK-8 assay kits. Firstly, BV-2 cells were seeded in the 96-well plates at a density of 10 000/well and incubated for 12 h. Subsequently, various concentrations of LPS (10–1000 ng/ml) or treatment drugs (free ARI and PA NPs) (ARI concentration of 0.5–20 μM) were added and co-incubated for another 24 h. Finally, the medium was discarded, and a fresh medium containing 10% CCK-8 reagent was added and incubated at 37°C for another 3 h. The absorbance values of each treatment group were detected at 450 nm using a microplate reader (Multiskan EX, Thermo Fisher Scientific, Waltham, USA). The cell survival was calculated as follow equation:

$$\mathrm{Cell}\;\mathrm{survival}\;(\%)={OD}_{\mathrm{sample}}/{OD}_{\mathrm{control}}\times 100$$

#### Inflammatory factors and NO release assessment

The inflammatory factors and NO release were determined using the corresponding ELISA assay kits and Griess reaction assay kit, respectively. The BV-2 cells were seeded in the 96-well plates at a density of 1 × 10^4^ cells per 100 μl per well and incubated overnight. The cells were pre-incubated with free ARI and PA NPs with an equivalent drug amount of 20 μM for 12 h. Then, all the experimental groups except the blank control group were incubated with LPS (1000 ng/ml) for another 24 h. Then, the cell medium supernatant was collected, and the release level of inflammatory factors and NO was determined using the corresponding assay kit.

### *In vivo* pharmacodynamic studies

#### Experimental design

The experimental procedures and timeline are shown in Fig. 6a. Male ICR mice (weight, 18–25 g) were randomly divided into control, LPS, LPS + ARI and LPS + PA NPs groups (*n* = 6). In our work, the designed composites were administered through the intraperitoneal injection. Briefly, the control and LPS treatment groups were injected with saline, LPS + ARI and LPS + PA NPs with a drug solution containing the same ARI content (0.8 mg/kg) daily for seven consecutive days. After 1 h of administration on the seventh day, all the experimental groups except the control group were further injected with LPS saline solution (1 mg/kg) to construct a depression-like mice model. Then, the anti-depressant negative symptoms effect of PA NPs was evaluated by various behavioral tests, including the sucrose preference test (SPT) and forced swimming test (FST). Finally, the mice in each group were sacrificed, and the blood and complete brain tissue were collected for further studies. During the administration period, the weight of mice was recorded every two days to assess the potential acute toxicity of the injected drug.

#### Sucrose preference test

The SPT was performed after the mice were deprived of food and water for 1 day. Firstly, the mice were given 1% sucrose solution and another bottle of purified water for free drinking for 24 h. The water consumption of each bottle was obtained by recording each bottle’s weight before and after the test and calculating the D-value. The percentage of sucrose preference was calculated as the following:

$$W\;\left(\%\right)=\frac{S_{c}}{T_{c}\times 100}$$

Where *W* was the sucrose consumption rate (%), *S*_c_ was the sucrose consumption, and *T_c_* was the total water consumption.

#### Forced swimming test

Firstly, mice were placed in clean buckets filled with water (room temperature) for 20 min of swimming training. At the formal FST, the mice were placed in the bucket for swimming for 6 min, and the immobility time of the last 4 min was recorded.

#### Immunofluorescence staining

Immunofluorescence staining was carried out through previous studies [51]. Briefly, the obtained brain tissue was fixed with 4% paraformaldehyde (PFA) at 4°C overnight and then dehydrated with a sucrose solution gradient. Then, the brain tissue was sectioned under freezing and further incubated with the antibody anti-Iba-1 (Abeam, USA) overnight at 4°C after blocking in 2% BSA. Subsequently, brain tissue sections were further incubated with secondary antibodies for 2 h at room temperature and the fluorescence image was observed under the confocal laser scanning microscope (CLSM, Leica TCS SP8, Wetzlar, Germany).

#### Determination of TNF-α level in mice serum

To further determine the effect of PA NPs on the production of inflammatory factors in mice, the expression of TNF-α in mouse serum was detected by an ELISA kit. Briefly, after behavioral tests, the mice of each experimental group, including the control, LPS, LPS + ARI and LPS + PA NPs group, were sacrificed by eyeball extraction, and the eyeball blood was collected in microcentrifuge tubes. Then, the blood samples were centrifuged, and the supernatant was collected to obtain the mice serum samples. Finally, the TNF-α levels in the serum of each group of mice were detected by the mouse TNF-α ELISA kit.

### Statistical analysis

Data were presented as mean ± standard deviations (SD). All experimental results were determined using the *t*-test analysis of variance at a defined significance value (*P* < 0.05).

## Results and discussions

### Optimization of PA NPs

**Figure 2.**
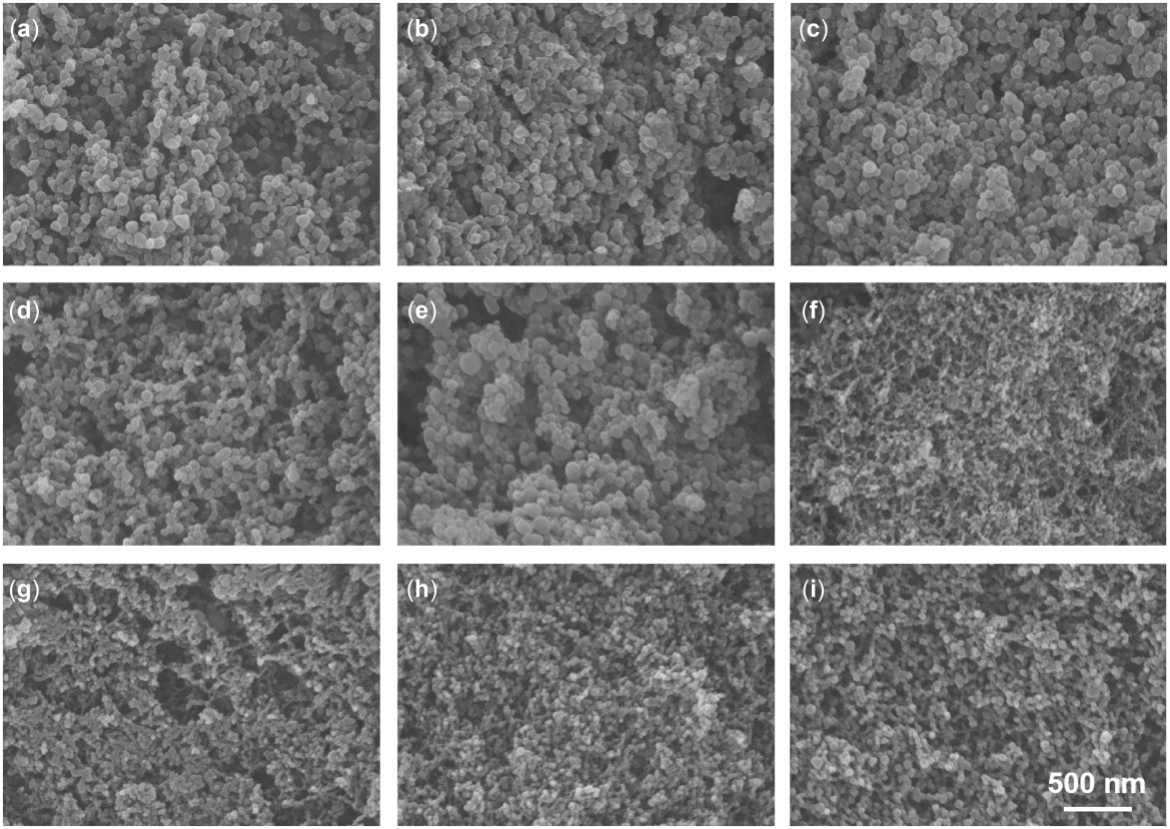
SEM Images of PA NPs prepared under different operating conditions (as shown in Table 1). (**a**) 1:10, 12, 0.5 ml min^−1^, (**b**) 1:2, 12, 0.5 ml min^−1^, (**c**) 1:10, 8, 1 ml min^−1^, (**d**) 1:10, 8, 0.5 ml min^−1^, (**e**) 1:10, 12, 1 ml min^−1^, (**f**) 1:2, 8, 1 ml min^−1^, (**g**) 1:2, 12, 1 ml min^−1^, (**h**) 1:2, 8, 0.5 ml min^−1^ and (**i**) 1:6, 10, 0.75 ml min^−1^ (the operating parameters are ARI/PVMMA mass ratio (w/w), pressure (MPa) and the flow rate of the solution mixture (ml min^−1^), respectively).

Initially, the homogeneous mixture solution of ARI/PVMMA (acetone as a solvent) was sprayed into an HPV filled with SC-CO_2_. Then, rapid phase transformation between mixture solution and SC-CO_2_ was achieved owing to the excellent performance of SC-CO_2_ (low viscosity and high diffusivity). Finally, the ARI/PVMMA mixture was precipitated into PA NPs under a high saturation state. The organic solvent was also eluted by the SC-CO_2_ flow (Fig. 1). A 2^3^ full-factorial experiment was designed to assess the effects of various parameters, including ARI/PVMMA mass ratio, pressure and flow rate, on the particle morphology (see Supplementary Table S1). Table 1 and Fig. 2 show the experimental results and the SEM images corresponding to each group of samples in the factorial design, respectively. According to Table 1 and Fig. 2, the SAS-assisted PA NPs showed particle sizes of 34.0–78.8 nm with different sphericity. Further, statistical analyses for the effect of various conditions on the morphology of the particles were carried out (see Supplementary Figs. S1 and S2). As depicted in Supplementary Fig. S1, the factor A (ARI/PVMMA mass ratio) and the interaction between factors A and C (flow rate) presented significant influences on the particle size. The mean particle size of PA NPs was positively correlated with ARI/PVMMA mass ratio and pressure and negatively correlated with flow rate. Supplementary Fig. S2 shows that factors such as factor B (pressure), the interactions between factor A and factor B, and the interaction between factor B and C, which significantly affect particle distribution. In addition, the changes in the span of PA NPs showed a positively correlated with these three factors.

**Table 1.** Mean size and span of PA NPs based on a minitab full-factorial design

Run order	Blocks	A	B	C	Mean size of PA NPs (nm)	Span (D90–D10)/D50
1	1	+1	+1	−1	62.6 ± 13.8	0.57
2	1	−1	+1	−1	63.7 ± 11.9	0.47
3	1	+1	−1	+1	72.9 ± 11.9	0.43
4	1	+1	−1	−1	65.6 ± 10.8	0.39
5	1	+1	+1	+1	78.8 ± 16.6	0.51
6	1	−1	−1	+1	34.0 ± 8.2	0.56
7	1	−1	+1	+1	38.7 ± 8.6	0.40
8	1	−1	−1	−1	40.7 ± 6.4	0.38
9	1	0	0	0	52.8 ± 8.3	0.37
10	1	0	0	0	55.7 ± 9.2	0.38
11	1	0	0	0	58.4 ± 7.7	0.41

**Figure 3.**
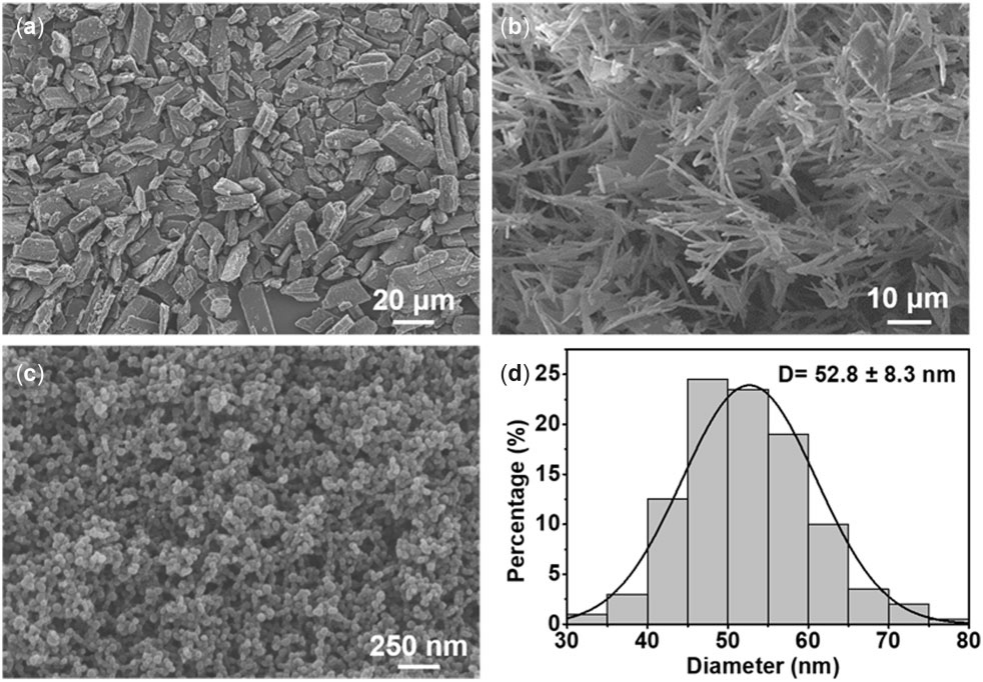
SEM images of (**a**) unprocessed ARI, scale bar = 20 μM, (**b**) SAS process-assisted ARI, scale bar = 10 μM, (**c**) PA NPs prepared under optimization conditions: ARI/PVMMA mass ratio of 1:6 (w/w), pressure of 10 MPa, solution flow rate of 0.75 mg ml^−1^, scale bar = 250 nm and (**d**) particle size distribution of PA NPs (*n* = 200).

Notably, the pure ARI showed an irregular block crystal (Fig. 3a). In contrast, the samples obtained after SAS treatment of pure ARI presented strip-like structures (Fig. 3b). In addition, it was observed that ARI alone could not be formed into nanoparticles with good sphericity by the SAS process in the absence of PVMMA. The results of the SEM observations and statistical analyses show that although these factors we investigated have a significant influence on particle morphology, the existence of PVMMA is the key reason for the formation of nanoparticles. The relevant mechanism had been explained in our previous studies [18], which might be due to interference of the polymer PVMMA with the crystallization kinetics of some drugs, leading to the co-precipitation of drug and polymer as nanoparticles. The preparation of PA NPs is a complementary process in which the addition of PVMMA promotes the nucleation of ARI in the SAS process and finally results in the formation of nano-sized particles. Based on the SAS process, further optimization of the experimental parameters was conducted to achieve accurate regulation of particle morphology. Accordingly, the optimal experimental conditions (ARI/PVMMA mass ratio of 1:6 (w/w), pressure of 10 MPa and solution flow rate of 0.75 ml min^−1^) were selected and used for the subsequent experiments. As shown in Fig. 3c and d, the PA NPs obtained under the optimal conditions exhibited uniform spherical, with a mean particle size of 52.8 ± 8.3 nm. Moreover, the drug loading efficiency of ARI in PA NPs under the optimal conditions was determined as 9.7% (see Supplementary Table S2).

### Characterizations

**Figure 4.**
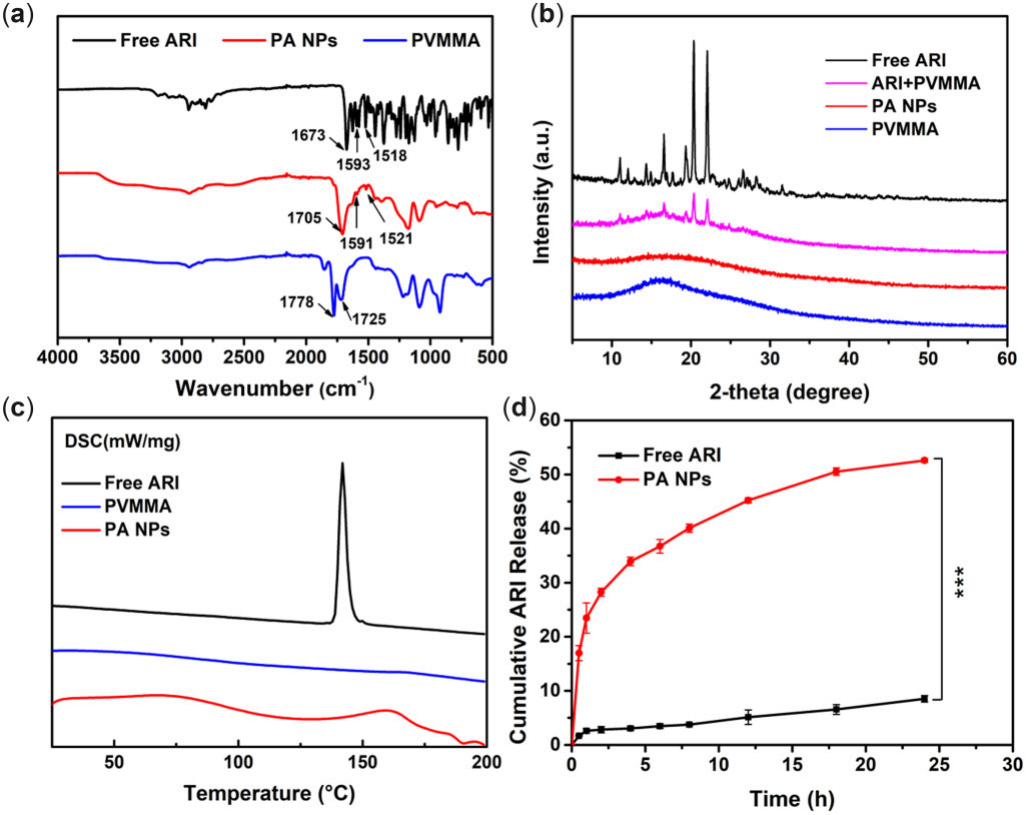
Characterization of physical and chemical properties of PA NPs. (**a**) FT-IR spectra of free ARI, PVMMA and PA NPs, (**b**) XRD patterns of free ARI, PVMMA, the physical mixture of ARI+PVMMA and the PA NPs, (**c**) DSC thermograms of free ARI, PVMMA and PA NPs, and (**d**) Cumulative drug release profiles of free ARI and PA NPs.

To verify the successful preparation of PA NPs, the FT-IR spectra of free ARI, pure PVMMA and PA NPs were recorded. As shown in Fig. 4a, the FT-IR spectra of free ARI showed some characteristic absorption peaks at 1673, 1593 and 1518 cm^−1^, which could be ascribed to C–O stretching, C = C stretching and –NO_2_ stretching, respectively. Concerning PVMMA, the presence of several characteristic absorption peaks representing PVMMA at 1778 and 1725 cm^−1^ could be attributed to the stretching vibration of the carbonyl group. The characteristic peaks of ARI and PVMMA could be observed in the spectrum of PA NPs, demonstrating the successful preparation of PA NPs by the SAS process. Moreover, a broad peak due to carbonyl stretching vibration appeared at 1705 cm^−1^ in the PA NPs spectrum, which could be due to the overlap of the nearby characteristic peaks of ARI and PVMMA. In addition, the intensities of the characteristic peak of ARI in PA NPs were much weaker than that of free ARI, which might be due to the lower ARI content in the samples. X-ray diffractograms of free ARI, pure PVMMA, the physical mixture of PVMMA + ARI and the PA NPs are shown in Fig. 4b. The free ARI showed four sharp diffraction peaks at 2θ values of 11.02°, 16.58°, 20.36° and 22.06°, indicating the crystallinity of the free ARI crystals. In contrast, the characteristic peak intensity of ARI was weakened in the physical mixture group, which might be attributed to the influence of amorphous PVMMA. Contrarily, the absence of the characteristic peak of ARI in the PA NPs group indicated that the ARI in PA NPs existed in an amorphous state. In addition, the XRD results were further verified by performing the DSC thermograms. As shown in Fig. 4c, in the thermal spectrum of PA NPs, the intrinsic melting absorption peak of ARI at 141.8°C becomes gentle to almost disappearing, indicating the amorphous physical state of ARI after the SAS process. The formation of amorphous ARI might benefit from the excellent mass transfer efficiency between SC-CO_2_ and the ARI and PVMMA mixture solution, resulting that the ARI molecules were having insufficient time to recombine into a crystalline structure during the formation of PA NPs [17, 52].

### *In vitro* dissolution study

The drug release behavior of PA NPs was evaluated by *in vitro* drug release test. As shown in Fig. 4d, the cumulative ARI release of free ARI was extremely low, about 8.5% in 24 h. However, the release ratio of ARI in the PA NPs group was significantly increased, about 52.6%. The improved drug release could be attributed to the SAS-assisted nano-sized PA NPs with a larger specific surface area and their amorphous physical state. In addition, PVMMA, as a polymer with good biocompatibility and aqueous solubility, might have played a crucial role in improving the solubility of ARI. Together, the presence of the water-soluble amorphous PVMMA might be the fundamental reason for the formation of nano-sized ARI in the SAS process.

### *In vitro* antidepressant effect

**Figure 5.**
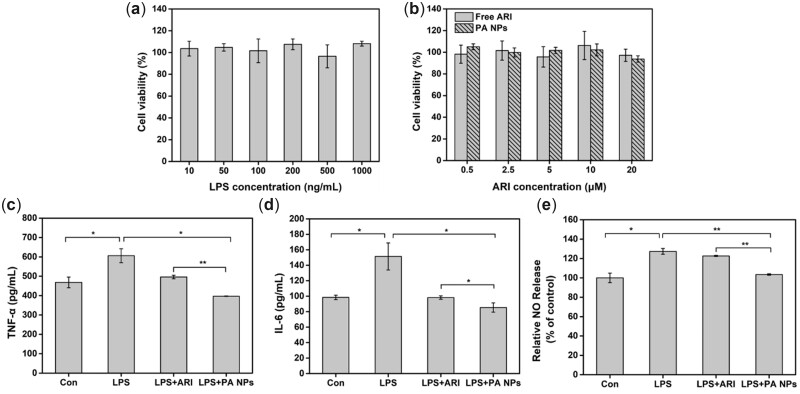
The anti-inflammatory effect of PA NPs on BV-2 cells. (**a**) Effects of different concentrations of LPS on BV-2 cell activity (LPS concentration of 10–1000 ng/ml), (**b**) effects of free ARI and PA NPs on BV-2 cell activity (drug treatment concentration of 0.5–20 μM). the effects of free ARI and PA NPs on the release of (**c**) TNF-α, (**d**) IL-6 and (**e**) NO in LPS-induced BV-2 cells (drug treatment concentration of 20 μM).

To eliminate the effect of cytotoxicity on drug efficacy, we first determined the safe dose concentrations of LPS and ARI by a CCK-8 assay. As shown in Fig. 5a, LPS showed no toxic effects on BV-2 cells in the concentration range of 10–1000 ng/ml at 24 h. Similarly, the activity of BV-2 cells was not significantly inhibited by PA NPs and the equivalent free-drug dose within the range of 0.5–20 μM in 24 h (Fig. 5b). The results demonstrated that LPS and ARI showed good cytocompatibility in the predetermined concentration range. Therefore, the maximum test concentrations of LPS and ARI for further study were 1000 ng/ml and 20 μM, respectively.

Patients with depression often exhibit an inflammatory response activation pattern. In this context, the activation of microglia was closely related to neuroinflammation induced by depression. In the process of neuroinflammation, microglia were activated after being stimulated, leading to the overproduction of various pro-inflammatory mediators (TNF-α, IL-6 and NO) [53, 54]. To evaluate the anti-neuroinflammatory effects of the PA NPs, an LPS-activated BV-2 cell line was used as an inflammatory model to study its inhibitory effect on TNF-α, IL-6 and NO. After LPS stimulation, the release of TNF-α, IL-6 and NO was significantly increased in the supernatant of BV-2 cells, which proved the successful construction of the inflammation model. (Fig. 5c–e). Regarding ARI and PA NPs treatment groups, especially the PA NPs group, the releases of these pro-inflammatory mediators were significantly reduced compared with the LPS treatment group, indicating that PA NPs showed a more effective anti-inflammatory effect (Fig. 5c–e). Collectively, these results suggested that the designed PA NPs could effectively improve the anti-inflammatory effect of ARI compared with the free ARI. These therapeutic effects could be improved due to the higher solubility and bioavailability of ARI in the prepared new nano-formulation (PA NPs).

### Behavioral tests

**Figure 6.**
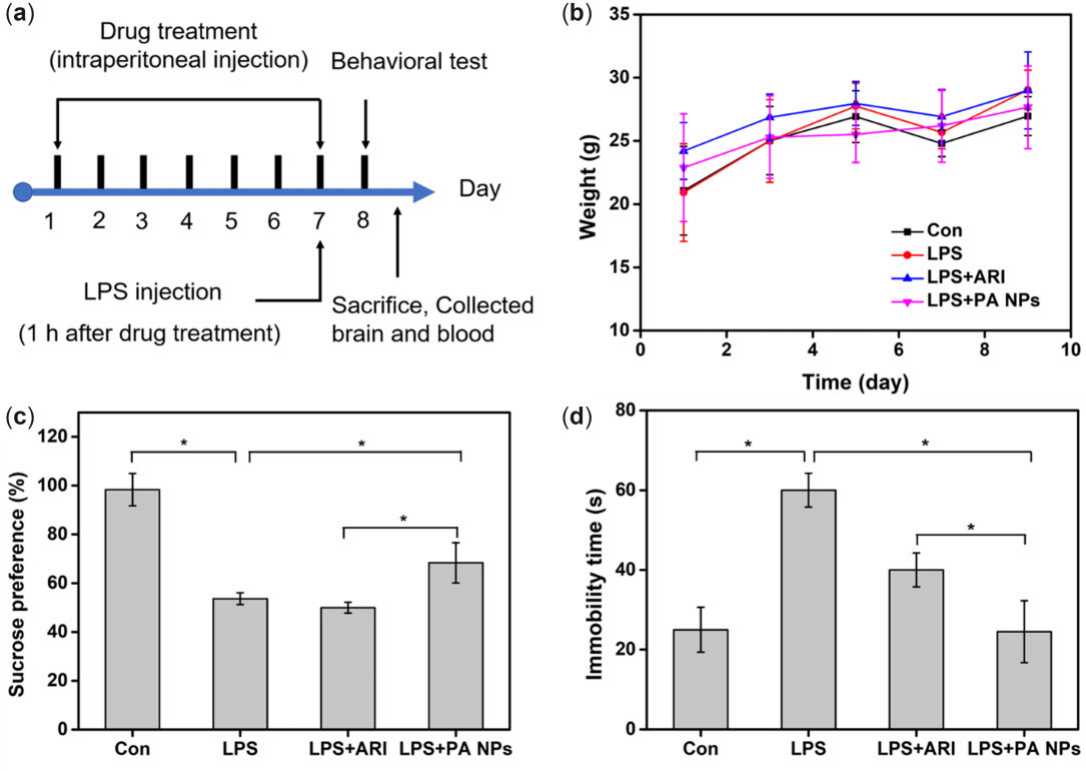
Effect of free ARI and PA NPs on treating depression-like mice. (**a**) Experimental procedures and timeline, the injection concentration of LPS and ARI were 1 mg/kg and 0.8 mg/kg, respectively, (**b**) Effect of free ARI and PA NPs on mice body weight changes. Depression-like behaviors tests of (**c**) Sucrose preference test (**d**) forced swimming test.

Furthermore, the effects of free ARI and PA NPs on LPS-induced depression-like behaviors in mice were investigated by various behavioral tests (SPT and FST). The body weight changes of mice in each group were recorded during the administration period (Fig. 6b). The results suggested that the weight of mice in each group increased slightly within 10 days without noticeable acute weight change, indicating that LPS, free ARI and PA NPs showed no apparent acute toxic and side effects on mice. In general, the negative symptoms of depression include anhedonia and hopelessness. SPT was usually performed to assess the anhedonia behavior in depressed mice. Compared with the control group, mice treated with LPS showed lower sucrose water intake. Compared with LPS group, the sucrose consumption of mice in the free ARI treatment group was decreased slightly, which may be due to the poor bioavailability of free ARI, resulting in almost no obvious therapeutic effect. However, after treatment with PA NPs, the sucrose consumption of mice was significantly increased (Fig. 6c). In addition to SPT, FST has been used to assess the degree of behavioral despair in mice by measuring the total immobility time. As shown in Fig. 6d, LPS treatment could significantly increase the immobility time of mice. In contrast, compared to the LPS group, PA NPs and free ARI treatment markedly reduced the immobility time (Fig. 6d). Importantly, PA NPs treated group showed lesser immobility time in comparison to the free ARI group (Fig. 6d). These results showed that PA NPs prepared by SAS process presented more obvious therapeutic effect on LPS induced depression-like behavior in mice compared with free ARI, which could be due to the improved bioavailability of ARI in the designed PA NPs in mice.

### *In vivo* anti-inflammatory study

**Figure 7.**
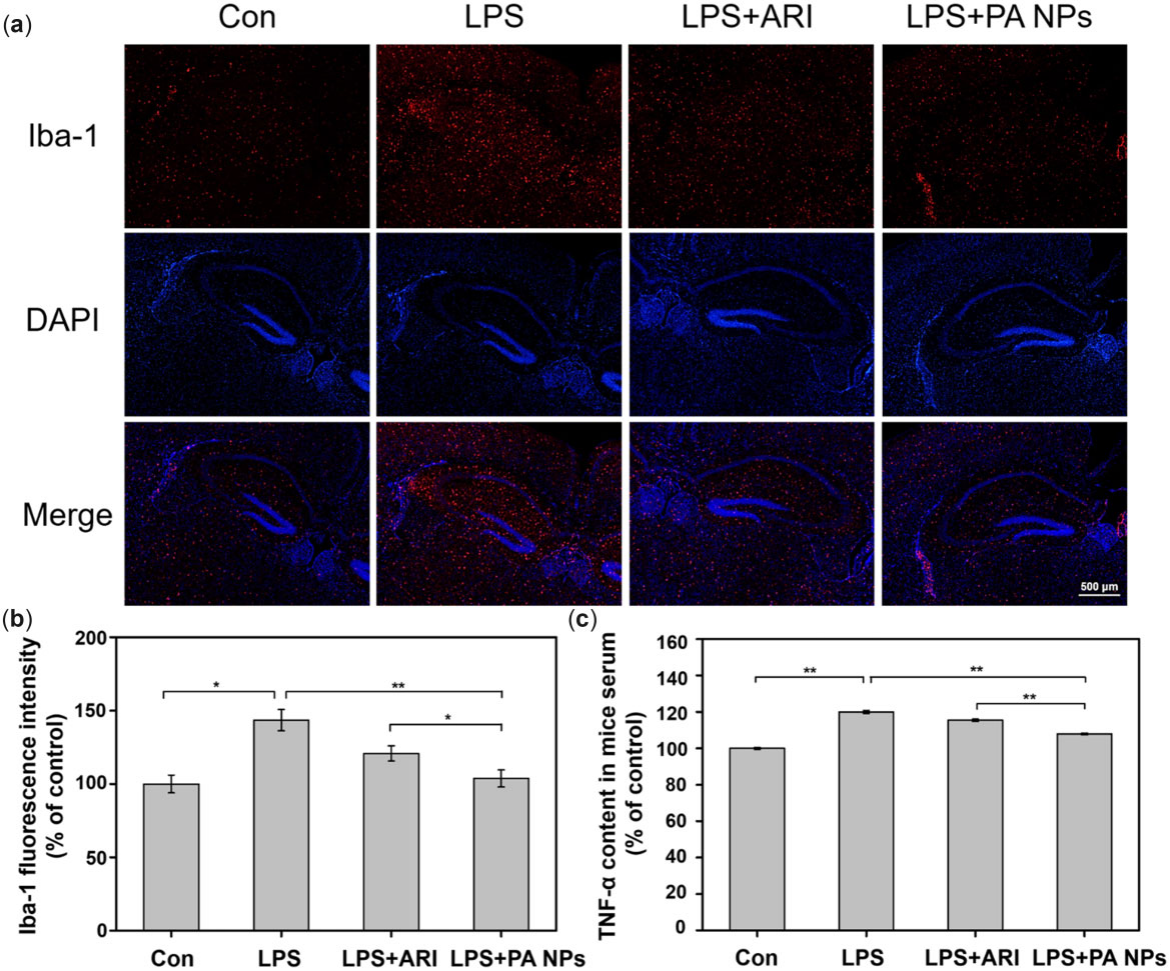
The effect of PA NPs preventing LPS-induced microglia activation and related inflammatory responses in mice. (**a**) Immunofluorescence images of iba-1 labeled microglia in the mice brain (red fluorescence represents the fluorescence signal of microglia labeled by iba-1, scale bar = 500 μM). (**b**) quantification results of iba-1 signal intensity. (**c**) PA NPs inhibited TNF-α expression in mice serum.

Microglia activation is often believed to be closely related to neuroinflammation induced by depression. Previous studies indicated that ARI could reduce the related inflammatory response by inhibiting the activation of microglia [55, 56]. To further measure the activation of microglia, immunofluorescence staining was used to observe the number of Iba-1 labeled microglia in the mice brain. It should be noted that Iba-1 is the commonly used microglia activation marker. Compared with the control group, the Iba-1 fluorescence signal (red) in the brain area of mice stimulated by LPS was significantly enhanced, indicating a large number of activation of microglia (Fig. 7a). In the mice injected with free ARI or PA NPs, Iba-1 fluorescence signals were dramatically decreased in broad brain areas, including the cerebral cortex, thalamus and hippocampus. Specifically, in the PA NPs group, the fluorescence intensity decreased to a level equivalent to the control group (Fig. 7a). Further, the quantitative results of fluorescence intensity quantified by ImageJ were consistent with the visually observed fluorescence results (Fig. 7b). These results suggested that PA NPs could significantly inhibit the activation of microglia induced by LPS in mice brain compared with free ARI. In addition, the TNF-α content was further detected in mice serum by ELISA kit (Fig. 7c). Similarly, PA NPs showed a more significant inhibitory effect on the increase of TNF-α expression induced by LPS. The results were consistent with the above-mentioned anti-inflammatory effects at the cellular level, as well as the behavioral test results. Together, it could be concluded that the SAS process-assisted PA NPs showed a better therapeutic effect on depression by inhibiting inflammatory reactions over the pure drug.

## Conclusions

In this study, PA NPs with excellent surface morphology were successfully prepared using the SAS process. The optimized experimental formula was obtained by a 2^3^ full-factorial experimental design. Moreover, the existence of PVMMA, a copolymer with good biocompatibility, significantly promoted the co-precipitation of ARI into nanoparticles. *In vitro* dissolution tests confirmed the PA NPs prepared by the SAS process showed an enhanced drug release due to their higher specific surface area and an amorphous physical state. Finally, the PA NPs showed potential anti-depressant efficacy by inhibiting the inflammatory response caused by LPS-induced microglia activation at the cellular and animal levels. In summary, the prepared PA NPs by SAS process might provide a feasible candidate for developing innovative ARI-based nano-formulations.

## Supplementary data

Supplementary data are available at *REGBIO* online.

*Conflicts of interest statement*. None declared.

## Funding

This work was supported by the National Natural Science Foundation of China (NSFC, 81971734, 32071323 and 32271410), and the Program for Innovative Research Team in Science and Technology in Fujian Province.

## Supplementary Material

rbac080_Supplementary_Data
