# Supplementary material for: Preparation of aripiprazole-poly(methyl vinyl ether-co-maleic anhydride) nanocomposites via supercritical antisolvent process for improved antidepression therapy
Source: Regen Biomater. 2022 Oct 12;9:rbac080. doi: 10.1093/rb/rbac080 (PMC9621701; doi:10.1093/rb/rbac080)
Supplement: rbac080_Supplementary_Data [file rbac080_supplementary_data.docx]

Supporting Information

**Preparation of Aripiprazole-Poly(methyl vinyl ether-*co*-maleic anhydride)** **Nanocomposites via Supercritical Antisolvent Process for Improved Antidepression Therapy**

**Lin-Fei Chen^1,2,3,†^,** **Ying Chen^1,2,3,†^,** **You-Yu Duan^1,2,3^,** **Man-Man Zhang^1^,** **Pei-Yao Xu^1,2,3^, Ranjith Kumar Kankala^1,2,3^, Shi-Bin Wang^2,3^, Ai-Zheng Chen^1,2,3,*^**

^1^College of Chemical Engineering, Huaqiao University, Xiamen, 361021, PR China

^2^Institute of Biomaterials and Tissue Engineering, Huaqiao University, Xiamen, 361021, PR China

^3^Fujian Provincial Key Laboratory of Biochemical Technology (Huaqiao University), Xiamen, 361021, PR China

^*^Corresponding author

E-mail address: [azchen@hqu.edu.cn](mailto:azchen@hqu.edu.cn) (A. Z. C.)

^†^These authors contributed equally to this work.

**Results**

**Table S1.** Experimental factors and levels of the Minitab analysis design.

| **Lever** | **Code** | **A**  **ARI/PVMMA Mass Ratio(w/w)** | **B**  **Pressure (MPa)** | **C**  **Flow (ml/min)** |
| --- | --- | --- | --- | --- |
| High lever | +1 | 1:10 | 12 | 1 |
| Center point | 0 | 1:6 | 10 | 0.75 |
| Low lever | -1 | 1:2 | 8 | 0.5 |


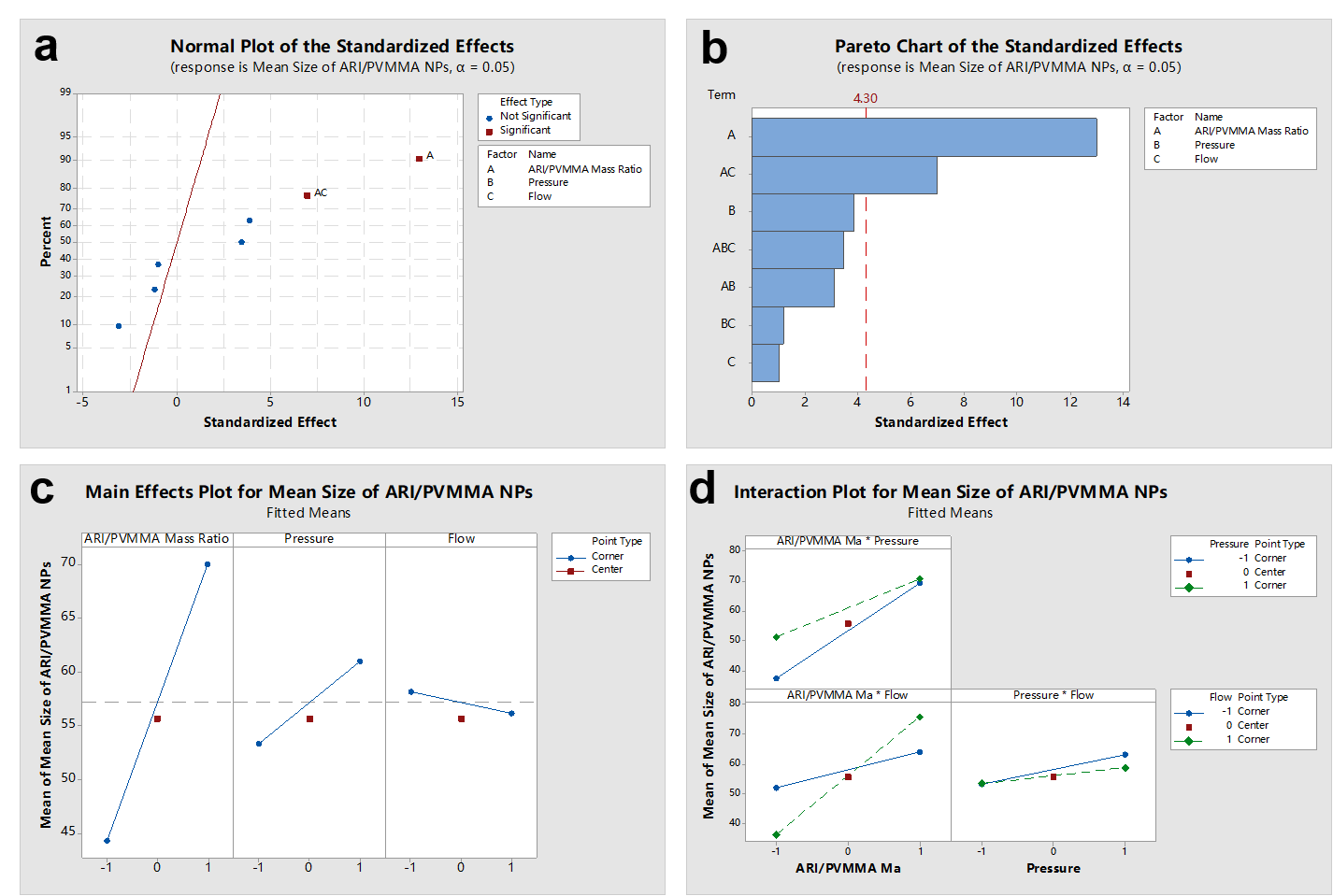


**Figure S1.** (**a**) Normal plot of the standardized effects of the factors on mean size, (**b**) Pareto chart of the standardized effects of the factors on mean size, (**c**) a main effects plot for mean size, and (**d**) an interaction plot for the mean size.


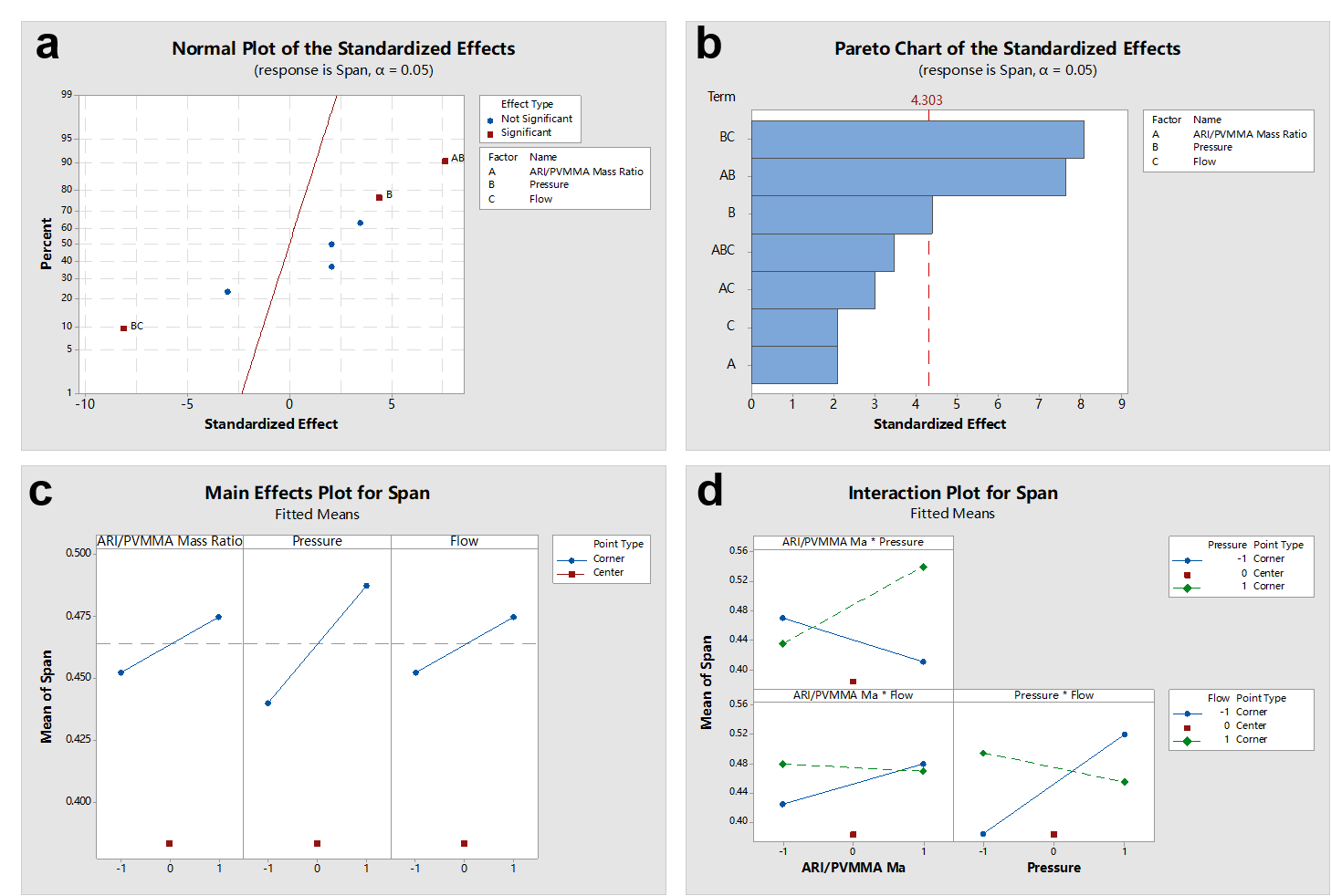


**Figure S2.** (**a**) Normal plot of the standardized effects of the factors on span, (**b**)Pareto chart of the standardized effects of the factors on span, (**c**) a main effects plot for span, and (**d**) an interaction plot for the span.

**Table S2.** The drug loading and entrapment efficiency of PA NPs in optimized group.

| ARI dosage | Drug loading efficiency | Encapsulation efficiency |
| --- | --- | --- |
| 14.3% | 9.7% | 68% |
